# Life during the pandemic: an international photo-elicitation study with medical students

**DOI:** 10.1186/s12909-021-02684-x

**Published:** 2021-04-27

**Authors:** M. Dworkin, T. Akintayo, D. Calem, C. Doran, A. Guth, E. M. Kamami, J. Kar, J. LaRosa, J. C. Liu, I. N. Pérez Jiménez, R. Frasso

**Affiliations:** 1 Warren Alpert Medical School, Brown University, Providence, RI USA; 2 College of Population Health, Thomas Jefferson University, Philadelphia, PA USA; 3 Obafemi Awolowo University, Ife, Nigeria; 4 Sidney Kimmel Medical College, Thomas Jefferson University, Philadelphia, PA USA; 5 Drexel University, Philadelphia, PA USA; 6 New Vision University School of Medicine, Tbilisi, Georgia; 7 Royal College of Surgeons in Ireland – Medical University of Bahrain, Busaiteen, Bahrain; 8 Universidad Nacional Autónoma de México, Facultad de Medicina, Mexico City, Mexico

**Keywords:** Global Health, COVID-19, Photo-elicitation

## Abstract

**Background:**

The SARS-CoV-2 (COVID-19) pandemic is a global event with unprecedented impact on individuals and communities around the world. The purpose of this study is to use a modified photo-elicitation methodology to examine the impact of the COVID-19 pandemic on the lives of medical students and their communities around the world.

**Methods:**

Participating medical students were asked to take photographs for 14 days. In lieu of an interview, which is customary for photo-elicitation projects, participants were asked to share a reflection (a paragraph or two) for each photograph they contributed to the study.

**Results:**

Between April 27th, 2020 and May 11th, 2020 26 students from 19 medical schools across 13 countries shared photographs and reflections. Qualitative analysis of written reflections revealed that medical students felt the impact of the pandemic on several levels 1) individual, 2) interpersonal, 3) educational, and 4) societal.

**Conclusions:**

The COVID-19 pandemic has impacted the lives of medical students on multiple levels. As individuals, students felt emotional distress but found resilience through physical activity and the establishment of new routines. Many students felt isolated as their interpersonal relationships were confined due to social distancing measures. These feelings could be combated with new educational initiatives focused on group collaboration. Lastly, students reflecting on the larger societal implications were concerned with the economic ramifications of the virus and its impact on their future. This study brought together students from several different countries to engage in an applied learning program as a model for equitable global health research.

**Supplementary Information:**

The online version contains supplementary material available at 10.1186/s12909-021-02684-x.

## Introduction

The SARS-CoV-2 (COVID-19) pandemic is a global event with unprecedented impact on individuals and communities around the world. The virus has infected over 127 million people and resulted in more than 2.8 million deaths as of March 30, 2021, with numbers increasing daily [1]. The novelty and scale of this crisis has overwhelmed health systems around the world and has led to the implementation of many public health interventions (PHI). These include the closing of schools and businesses, mandating the use of face masks in public, and the implementation of social distancing standards [2].

Few events in recent history have disrupted society as much as the COVID-19 pandemic [3]. While PHI have been shown to be effective to varying degrees, they have resulted in complex societal changes and challenges [4, 5]. For example, unemployment in the USA reached 14.7% in April of 2020 with the loss of 20.5 million jobs [6]. Schools and universities have closed in 192 countries, disrupting the education of nearly 90% of students around the globe [7]. These changes have had large economic ramifications and have contributed to a rise in prejudice, xenophobia [8] and social unrest. Related protests around the world have led to additional public health challenges.

Even as the world, slowly reopens, understanding the impact of the pandemic on individuals and communities is an important first step on the road to recovery from the physical, emotional, social and economic toll it has taken around the globe. Studying the lived experience of individuals during disasters can help improve recovery and prepare for future events [9–11]. As the pandemic unfolded, medical students had an important view of the impact of the COVID-19 pandemic on themselves and the communities they were trained to serve [12]. Medical education has changed drastically with limitations on in person learning preventing many from engaging in clinical rotations [13]. Additionally, online learning has become the primary teaching model which has posed challenges for students without access to reliable internet and teachers without experience using online mediums [13, 14].

This study utilized a modified photo-elicitation approach [15]. Briefly, photo-elicitation asks the participants to generate and to share photographs that would help them explain an experience, event or feeling [16, 17]. The approach has been shown to be effective in the exploration of important issues, which may be difficult to discuss in typical interviewer-led questioning [18, 19]. The purpose of this study was to examine the impact of the COVID-19 pandemic on the lives of medical students and their communities around the world by using a modified photo-elicitation approach.

## Methods

### Study overview

This study’s protocol was approved by the Institutional Review Board at Thomas Jefferson University. All methods were carried out in accordance with relevant guidelines and regulations. Unlike a quantitative study, which uses deductive reasoning to test a theory on the basis of predetermined domains, in this qualitative study, we used an inductive approach to uncover an understanding on the basis of the experiences and vantage point of medical students from 13 countries during the early months of the COVID-19 pandemic. In particular, the study was designed to explore the experiences and perceptions held by these students as they reflected on how the pandemic impacted them and their communities. The study was not intended to quantify or weight responses in any way. The study design was informed by a comprehensive literature review and expert consultation [20]. Participating medical students were asked to take photographs for 14 days. In lieu of an interview, which is customary for photo-elicitation projects, participants were asked to share a reflection (a paragraph or two) for each photograph they contributed to the study.

### Research team

In an effort to incorporate an international and cross disciplinary perspective, a team of medical students from around the globe was assembled by the first author (M.D.) and their work was supported by three Master’s in Public Health (MPH) students (J.L., A.G., and C.D.) and supervised by a qualitative researcher with over 15 years of experience (R.F.). The entire team met virtually during the data collection, coding, and analysis phases of the study.

### Participants

Medical students from around the world were recruited for involvement in this study. Participants were eligible for enrollment if they were currently matriculated at an institution and pursuing a doctorate in medicine or their respective country’s equivalent. Although not a requirement for inclusion, all participants were fluent in English. Study participants were recruited through email invitation via the Global Educational Exchange in Medicine and the Health Professions (GEMx) network. GEMx is an Educational Commission for Foreign Medical Graduates (ECFMG) sponsored program that helps medical students connect around the world and it has partnerships with 56 medical schools in 31 countries. The first email invitation was open for 1 week or until 50 volunteers reached out, whichever came first. A constant-comparison approach was used, whereby reflections were reviewed and open coded as they arrived. On the basis of this information, a sample size of 26 was considered sufficient to achieve saturation, meaning that later reflections did not generate new codes [21, 22]. The sample population was a convenience sample, and no comparisons were made between participants in the sample population and other medical schools. All participants were provided with study details and electronically signed consent prior to enrollment in the study.

### Data collection

Participants were asked to take 3–4 photographs over a two-week period depicting daily life during the pandemic, and then write brief reflections about each photograph. Photography allows participants to process and experience as they prepare to share their thoughts with a research team through interviews or, as in this case, written reflections [17]. Photography, as a qualitative elicitation tool, has also been shown to improve participants’ memories and elicit longer and more comprehensive narratives about an experience [23]. Participants were asked to record pictures of their normal lives and not to seek out stressful experiences, take any risks or deviate from their normal routines [16]. Due to ethical concerns, photographs of individuals other than the participant were not accepted unless permission was granted by the third party. All participants captured photographs using their own devices and gave explicit permission for the photographs to be used in publications or exhibits. Additionally, participants were asked to complete a brief demographic survey, share details about their university, and to reflect on how COVID-19 has impacted them as well as their communities (Survey provided as supplementary file). Data were collected using Research Electronic Data Capture (REDCap) [24].

### Photography

Participants were asked to record the date each picture was taken, note a general location. and write a brief reflection. In their reflections, participants were given freedom to discuss any topics they associated with the photographs.

### Analysis

A team of medical students from 6 countries, as well as three MPH students from the US, were assembled to code and conduct data analysis. A directed content analysis approach was used [20]. Briefly, the reflections from the first five participants were open coded by several research team members (M.D., J.K., J.L., N.P., and T.A.), then the entire international team met virtually to discuss possible codes, generate a draft code book and explicit coding definitions. The code book was piloted on a sample of reflections by multiple members of the team (M.D., J.K., N.P., T.A., and E.K.). The entire team met again to finalize the codebook. Data were transferred to NVivo12 software and coded independently by three members of the team (J.L., A.G., and C.D.). Intercoder reliability was assessed using the kappa statistic function in the program. Coding discrepancies were resolved by consensus at team meetings.

### Reliability

Intercoder reliability was calculated using NVivo12 software and the kappa (*k*) coefficient. The *k* coefficient compares agreement and disagreement between coders. A *k* value of 1 represents complete agreement between coders; 0.81–0.99 represents near perfect agreement, 0.61–0.80 represents substantial agreement, and 0.41–0.60 represents moderate agreement. An examination of intercoder reliability for this data set revealed substantial agreement (mean *k* = .99 across all codes; range, 0.79–1.00).

## Results

### Explanatory model development

**Fig. 1:**
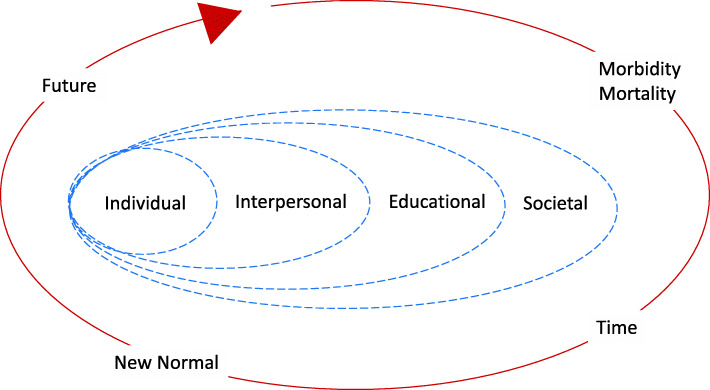
Explanatory model based on content analysis demonstrating various levels of influence with overarching themes

After coding was complete, a subset of the team met (M.D., R.F., J.L., A.G.), reviewed coding output, and organized the findings into thematic categories (Table 1) to inform an explanatory model (Fig. 1).

**Table 1:** Grouping of themes into levels of influence

*Thematic Categories*	
Level	Codes
Individual	Feelings
	Physical health
	Mental health
Interpersonal	Family
	Environment
	Living situation
	Communication
	Support
Educational	Education
	Training
	Technology
Societal	Country
	Services
	Economic issues
	Restrictions and Safety
	Healthcare
	Technology

### Demographics

Between April 27th, 2020 and May 11th, 2020 twenty-six students from 19 medical schools across 13 countries shared photographs and reflections (Table 2). The average age was 24 (standard deviation 4) and 14 (54%) were male (Table 3). Most students were in their 4th or 5th year of medical school. The predominant model for education during the pandemic was through online learning classes. 10 (35.5%) students relocated due to the pandemic and 18 (64%) students were forced to make changes to their academic timeline. Other demographics of participating students are described in Table 3.

**Table 2:** List of countries represented in study

Countries	Number of Students
Dominican Republic	1 (4%)
Egypt	1 (4%)
Georgia	1 (4%)
India	6 (23.0%)
Ireland	4 (15.0%)
Kyrgyzstan	1 (4%)
Mexico	1 (4%)
Nigeria	4 (15.0%)
Pakistan	1 (4%)
Rwanda	1 (4%)
South Africa	1 (4%)
Uganda	2 (7.5%)
United States of America	2 (7.5%)

**Table 3:** Demographic and school information of study participants

Demographics	Number of Students (%)
Age (Standard Deviation)	24 (+ 4)
Sex	
Male	14 (54%)
Female	12 (46%)
Year of Medical School	
1st	0 (0%)
2nd	4 (15.5%)
3rd	1 (4.0%)
4th	7 (27%)
5th	9 (34.5%)
6th	3 (11.5%)
Students forced to relocate due to pandemic	10 (35.5%)
Primary model of education during pandemic	
In person class	1 (3.5%)
Online class	19 (68%)
Classes cancelled or semester holiday	8 (28.5%)
Change in academic timeline	18 (64%)

### Thematic interpretation

Qualitative analysis of written reflections revealed that medical students felt the impact of the pandemic on several levels 1) individual, 2) interpersonal, 3) educational, and 4) societal. Additionally, they recognized, documented and reflected on the burden it inflicted on the communities around them. The impact at every level was influenced by overarching factors, which included a) acknowledging and adjusting to the “new normal”, b) a desire or inability to imagine the “future”, c) the passage of “time”, and d) the reality that the virus was impacting “morbidity and mortality” constantly. In Table 1, we share the codes that fell into each category and below we share summative statements about each supported by excerpts from participant reflections, as well as selected participant photographs (de-identified as needed). All photos selected for this manuscript were used with permission from study participants.

### Individual level

**Fig. 2:**
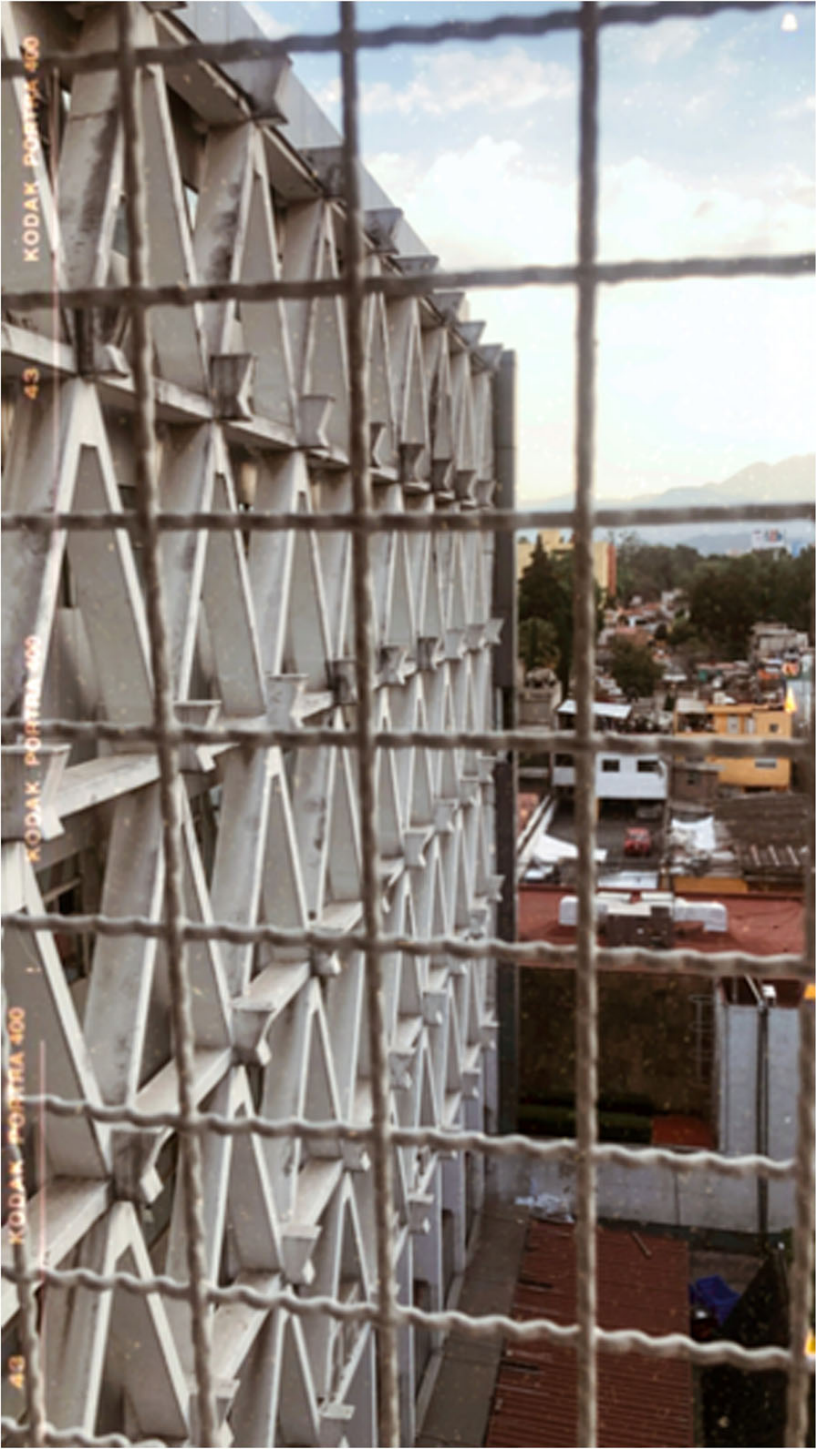
Photo taken by female in Mexico City, Mexico of her stair view

**Fig. 3:**
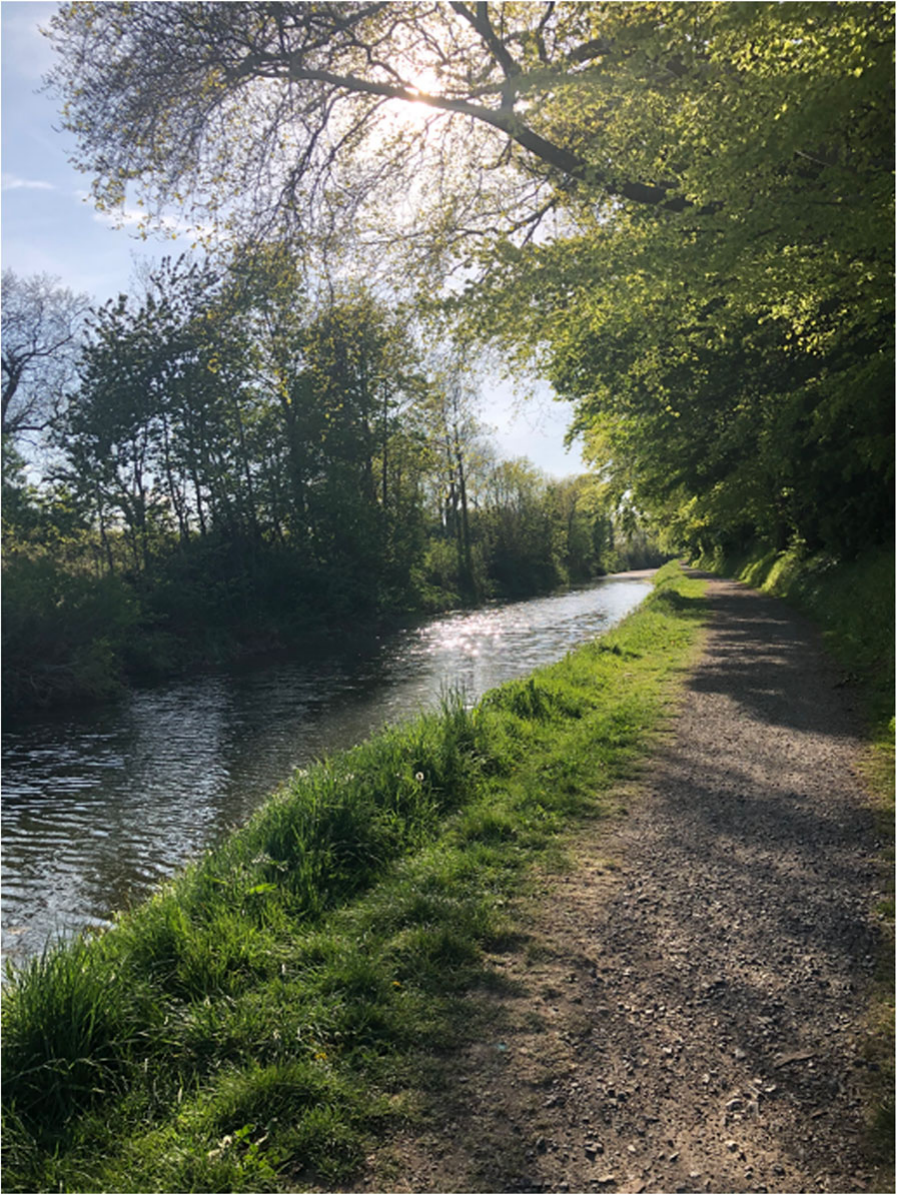
Photo taken by a female in Dublin, Ireland of her nature scenery

Participants explained that the pandemic had an impact on each of them as individuals, students, and members of the community. They shared their feelings about the pandemic and its impact on their physical and mental health. Participants explained that they grappled with a multitude of personal feelings, including fear, grief, and denial. They also expressed a desire to address these feelings by giving themselves “a break,” “stay [ing] positive” and taking care of themselves despite what was happening (Figs. 2 and 3).

*“Fear dominates when you come out of your house.” (Male – Santo Domingo, Dominican Republic)*.

*“I felt very overwhelmed and decided to give myself a break and go to the stairs and breathe a little bit of fresh air.” (Insert Fig.* *2**here, Photo taken by female in Mexico City, Mexico of her stair view.)*

*“So, I would say that in this pandemic I would like to stay positive and work on myself rather than complaining of things I do not have control over!” (Female – Tbilisi, Georgia).*

*“I am lucky to live in a place that’s surrounded by nature. I love to walk on the canal daily to escape from being in the same place all day. This gives my mind a break and gives me some time to myself.”* *(Insert Fig.* *3**here, Photo taken by a female in Dublin, Ireland of her nature scenery.)*

**Fig. 4:**
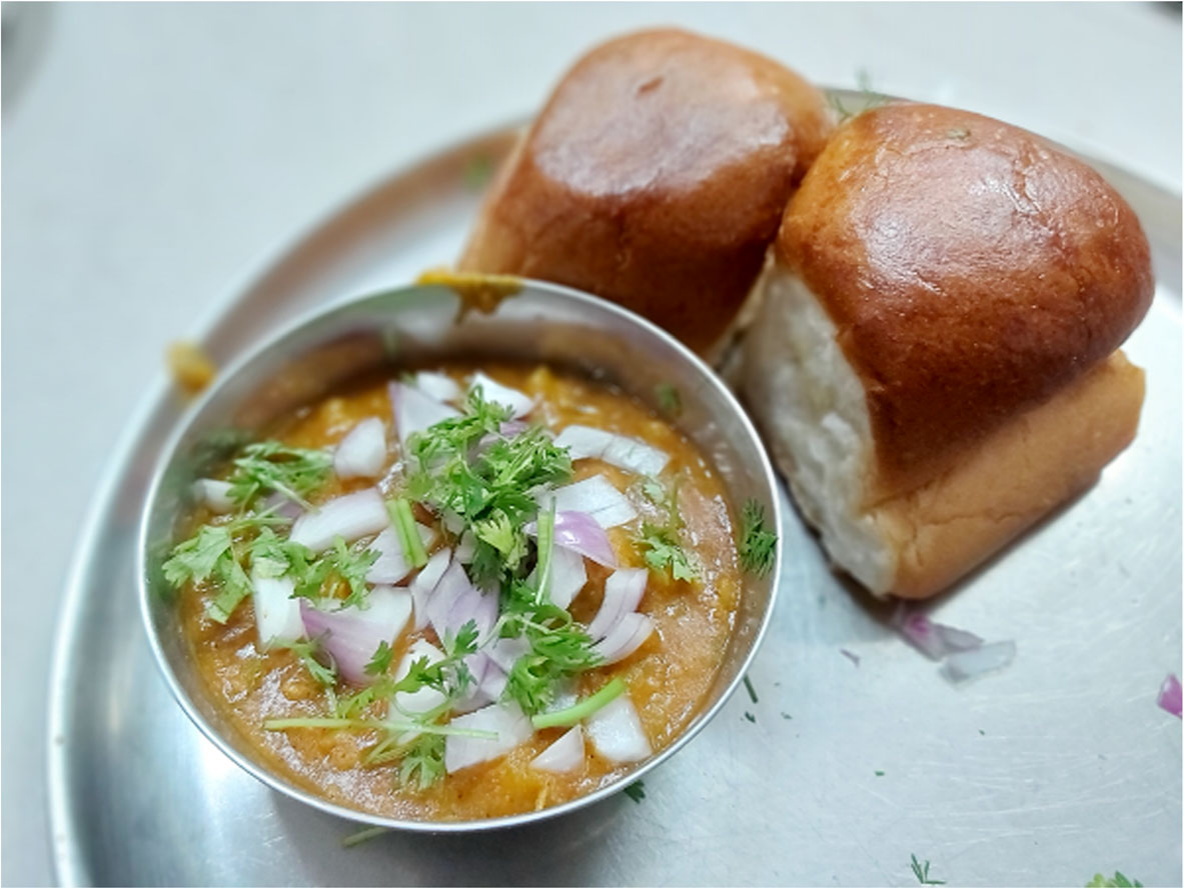
Photo taken by a female in Pune, India of her “pau bhaji,” a famous Indian dish

In addition to managing a plethora of emotions, many participants described how they adapted their routines to create space and time for selfcare. Students are finding new ways to engage in physical exercise and engaging in new hobbies. One student described their attempt to learn a new skill while maintaining a balanced diet during COVID-19 (Fig. 4).

*“I have been learning new dishes since there is lot of time for learning new things. The photograph here is a new dish I learned called “pau bhaji”, it’s a famous Indian street food.”* *(Insert Fig.* *4**here, Photo taken by a female in Pune, India of her “pau bhaji,” a famous Indian dish.)*

### Interpersonal level

Participants reflected on how the pandemic modified the way in which they interact with friends, family and others in their community. Many noted that their living situations and daily routines had to be adjusted. The pandemic led to separations from friends or partners and small losses related to not being able to share spaces and experiences with others.

*“It has been very frustrating having to be separated from [my girlfriend] for so long since we have been together since coming out of high school. We went through medical school together, seeing each other every day. This is hard to explain, but I enjoy vastly seeing her at the library or hospital, so having her stripped away for weeks hasn’t felt nice.” (Male – Santo Domingo, Dominican Republic).*

Additionally, many respondents noted that the lack of interpersonal communication and interaction has had an impact on their behavior. There was concern for potential long-term effects on one’s emotional well-being. Participants noted ways to stay connected and how to assure others were doing well.

*“The worst part of ourselves can come out when confinement extends for so long.” (Male – Santo Domingo, Dominican Republic).*

*“One of the best ways for me to stay both sane and academically accountable during this time has been through Zoom study sessions with my study buddies.” (Female – Bloomington, United States of America).*

There were, however, some unexpected benefits to being sidelined during the pandemic.

For example, some students expressed having more time with their family.

*“For me, this is the first time since I left for boarding school that I have sat down for consistent family meals. I’ve finally been able to catch up on my siblings’ and parents’ personal lives and understand more about their day to day.” (Male – Philadelphia, United States of America).*

### Educational

**Fig. 5:**
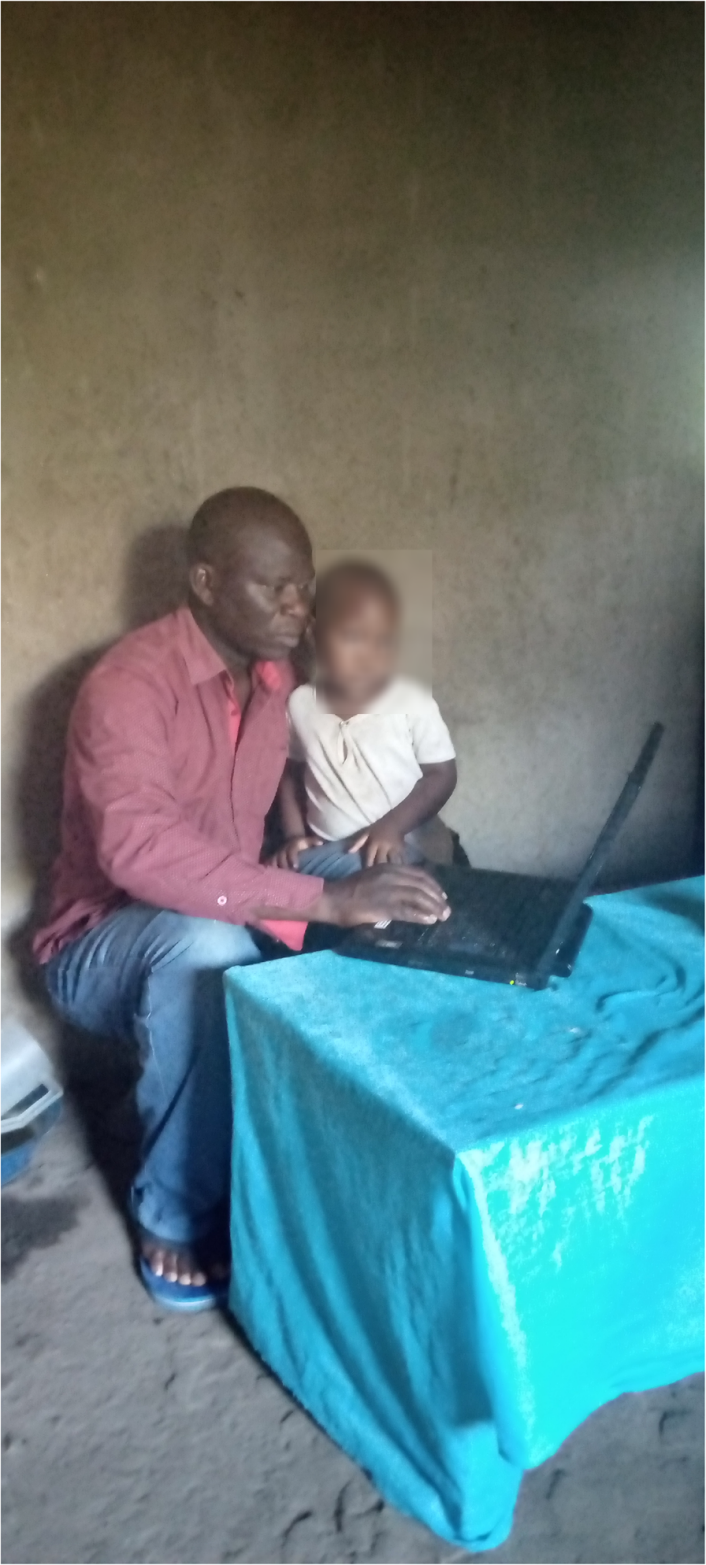
Photo taken by a male in Kable, Uganda of his workspace during lockdown

**Fig. 6:**
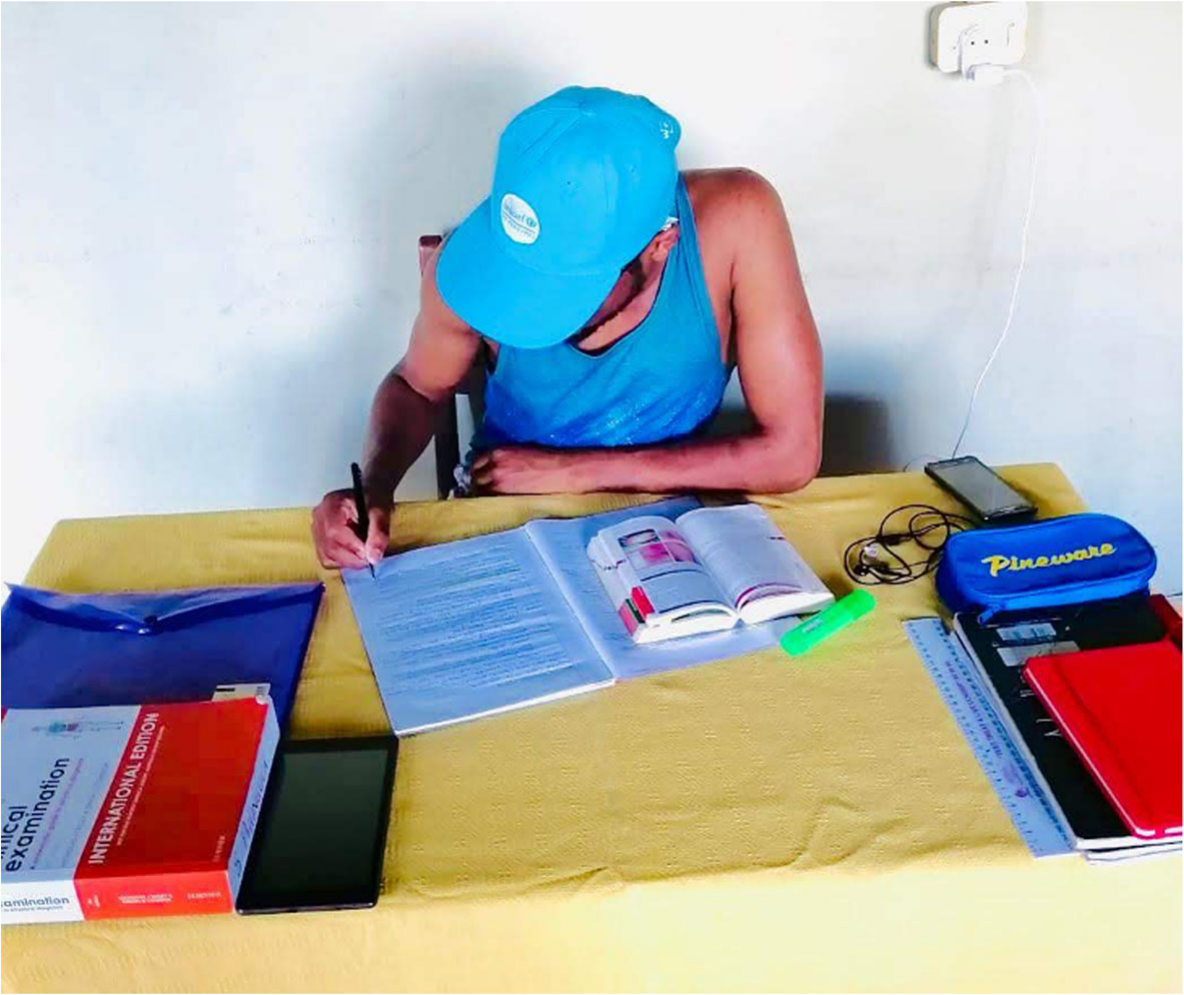
Photo taken by a male in Durbin, South Africa of his workspace during lockdown

Students shared that education, like everything in life, was impacted by the pandemic. Many respondents described struggling with technology and how shifts to virtual platforms often interfered with fulfilling personal responsibilities (Figs. 5 and 6). Additionally, participants explained how they leveraged technology to stay connected with classmates and support one another.

*“Taking into consideration the challenges of poor network connectivity and not having devices to access study materials is challenging to students as a whole [as we have to] juggle with outside responsibilities alongside our schoolwork.” (Insert Fig.* *5**here, Photo taken by a male in Kable, Uganda of his workspace during lockdown.)*

*“Lockdown restrictions meant contact classes cannot continue and thus universities across the country adopted online learning. That meant, I had to work from home which has been quite a challenge on its own. I try to put at least 6 hours study time a day and sometimes that is impossible due to the conduciveness of the study space. I therefore work at night, because there are less distractions and I am more productive then.” (Insert Fig.* *6**here, Photo taken by a male in Durbin, South Africa of his workspace during lockdown.)*

*“It has been one of the highlights of my day to get to sit down and talk to my friends from all over the world while we all go through the same things.” (Female – Bloomington, United States of America).*

Many of the participants described how their universities or institutions increased efforts to teach staff and students about COVID-19 and how to stay safe.

*“On this day I was asked to go to a course to learn how to use the personal protection equipment so that when the quarantine is over, I can be prepared to go to my hospital. This represents the effort of the university to prepare us students.” (Female – Mexico City, Mexico).*

Several participants were still spending time in a clinical setting. They described how.

their workload was impacted by decisions made to reduce staff and student exposures.

*“One afternoon we had too much work to do and because of the [staff were released] to help reduce the number of people in contact with the potential COVID cases, we were only 4 students and the residents doing the work.” (Female – Mexico City, Mexico).*

### Societal level

**Fig. 7:**
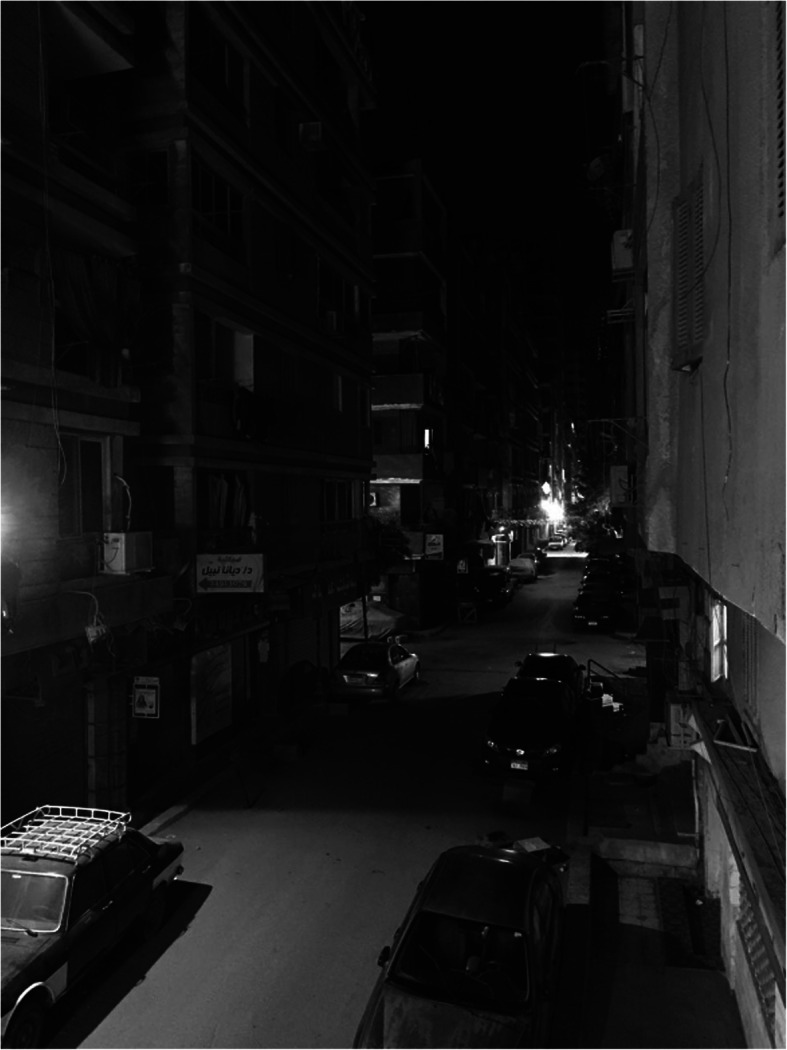
Photo taken by a male in Cairo, Egypt of a quiet street in Cairo

**Fig. 8:**
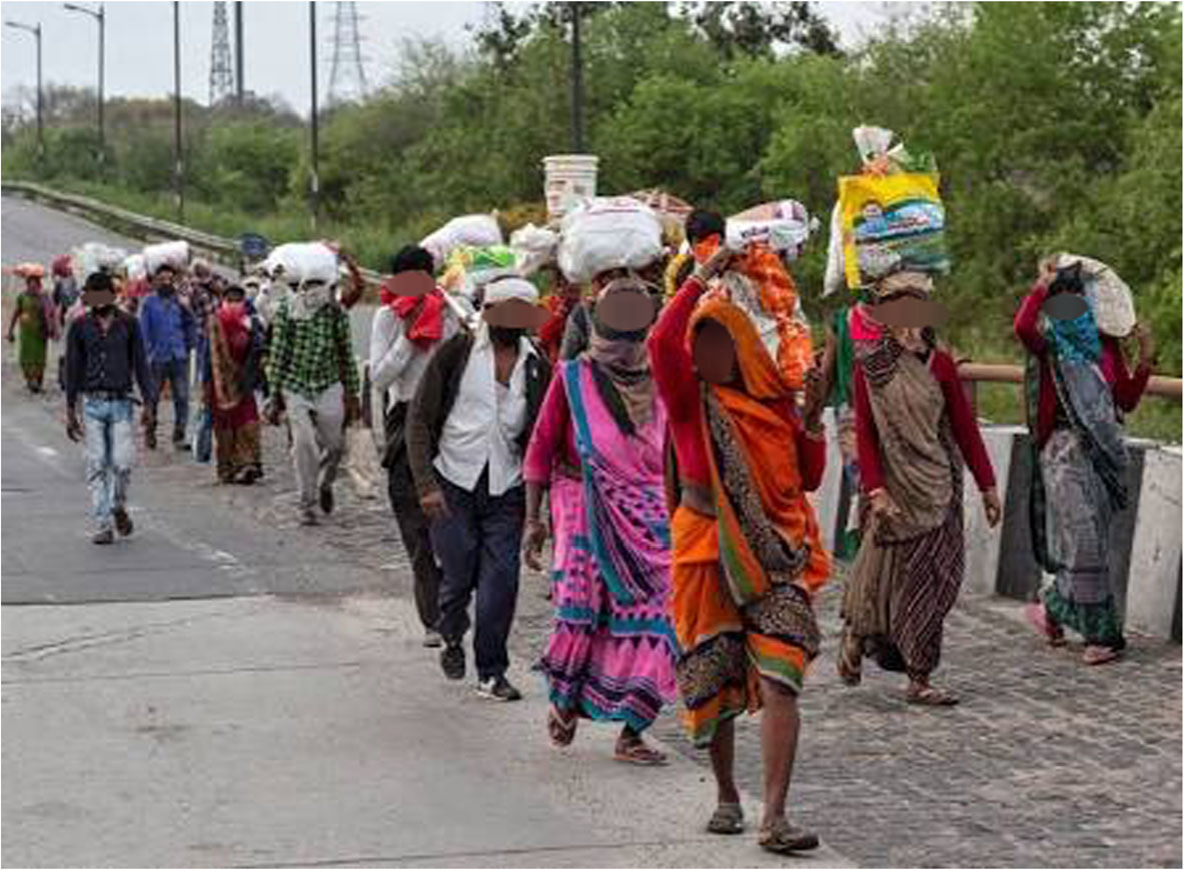
Photo taken by a female in Mumbai, India of workers in India

Respondents documented and reflected on how the pandemic led to shifts in society. They noted observable changes in human movement and activity, as well as in national policies. Participants discussed the impact of the pandemic on economies, public services, educational platforms, training opportunities, healthcare environments, technology, and rules regarding restrictions and safety. For example, many participants noticed the decrease in activity in their respective communities and shifts in population. Participants expressed concern about the impact this could have on the economy (Figs. 7 and 8).

“Silence everywhere in the streets. Cairo, the city that is called "the non-sleeping city" is now empty!” (Insert figure 7 here, Photo taken by a male in Cairo, Egypt of a quiet street in Cairo.)

*“These are a group of migrant workers walking to go back to their homes, which include small children, pregnant women, etc., from the place where they came to work in a major metro city of India. These workers are mostly daily wagers but due to lockdown which was announced on the 24*^*th*^ *of March led to closure of all large and small scale industries in India. This has put workers into deep crisis as their source of income halted.”(Insert Fig. * *8**here, Photo taken by a female in Mumbai, India of workers in India.)*

They also spoke about the impact of the media on the wellbeing of communities and the government’s role in protecting society.

*“But what I notice is that the fear that the media and some health workers sell is more killing than the pandemic itself. One of the core responsibilities of the government and health workers is to allay fear even when the media is doing the contrary because fear and panicking can cause a lot of negative effect for the public's health especially people with anxiety disorders. Some people have died not because of the outbreak but because of the neglect and fear that was presented.” (Male -Ibadan, Nigeria)*

**Fig. 9:**
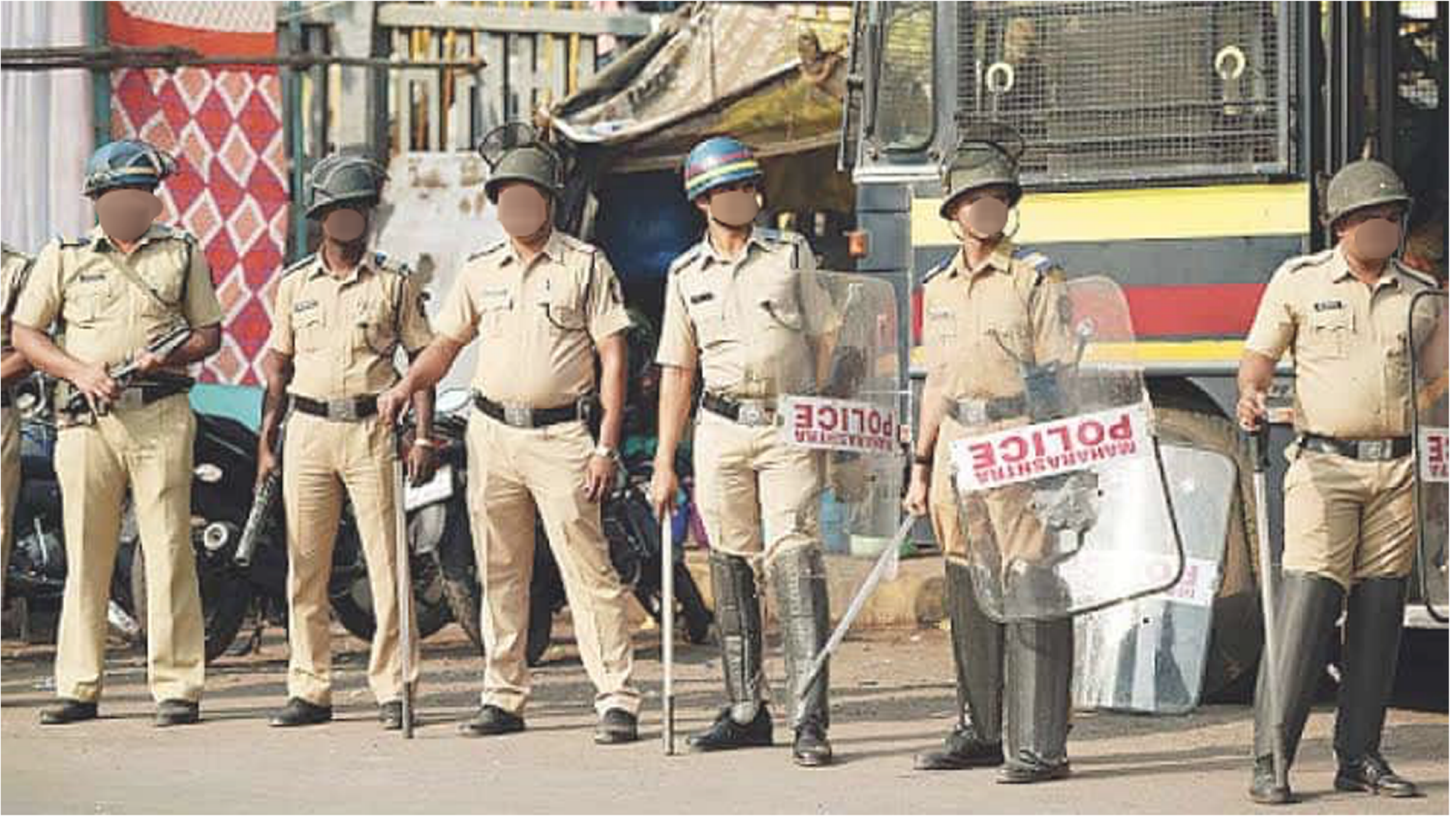
Photo taken by a male in Mumbai, India of police in India

**Fig. 10:**
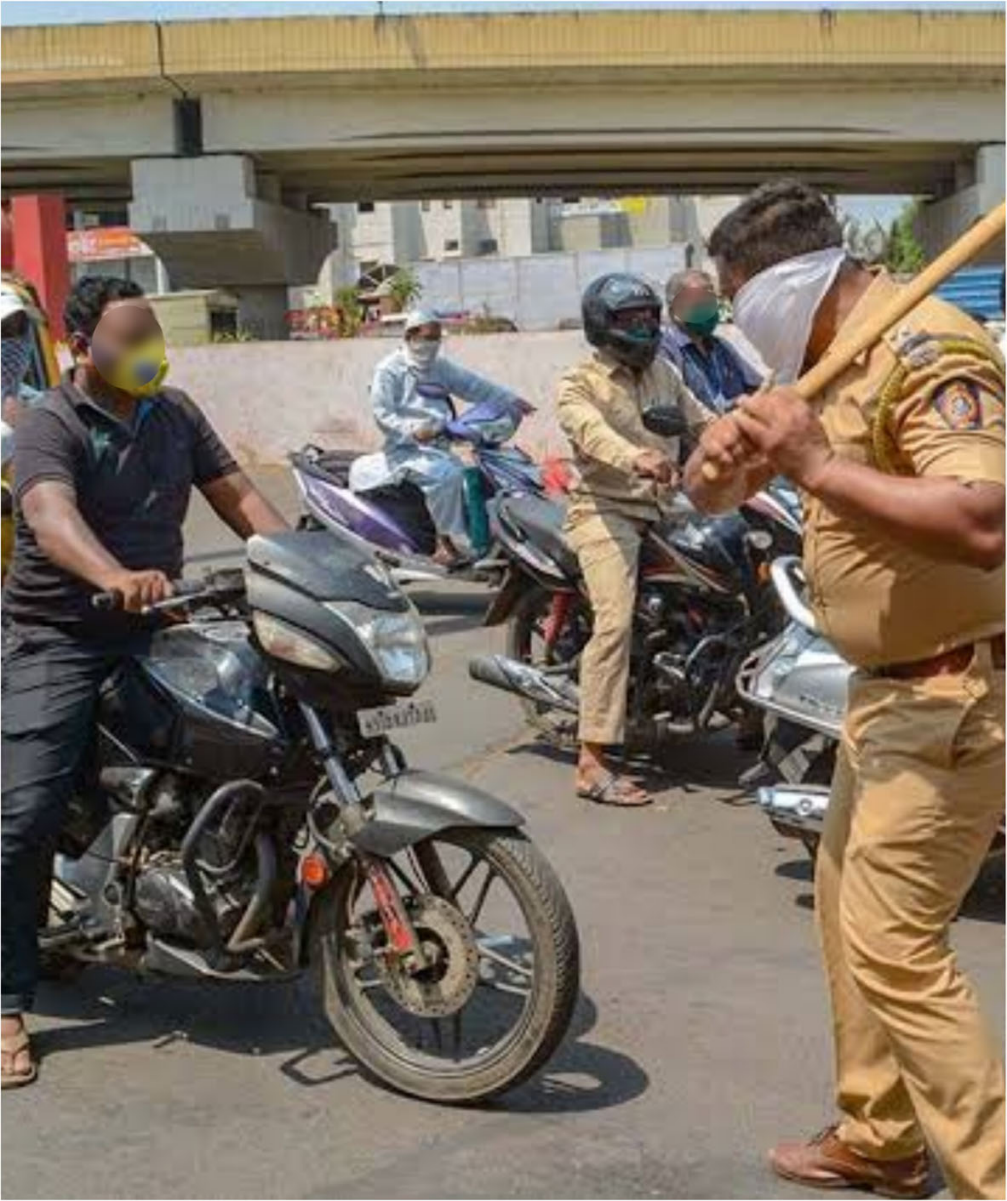
Photo taken by a male in Mumbai, India of police taking action against citizens who are not abiding by lockdown security measures

Various views were expressed regarding the government’s response to the pandemic. Police and military have been on the frontlines tasked with enforcing restrictions (Figs. 9 and 10).

“Mumbai has become the epicentre of virus in India infecting more than 19000 people. This is a group of policemen of Maharashtra police who are frontline warriors along with health personal such doctor and nurse. The police have been allotted the duty to maintain discipline during the crisis of COVID19.” (Insert figure 9 here, Photo taken by a male in Mumbai, India of police in India.)

“There have been several reports of police beating people with lathis and ensuring public security.” (Insert figure 10 here, Photo taken by a male in Mumbai, India of police taking action against citizens who are not abiding by lockdown security measures.)

### Overarching factors

**Fig. 11:**
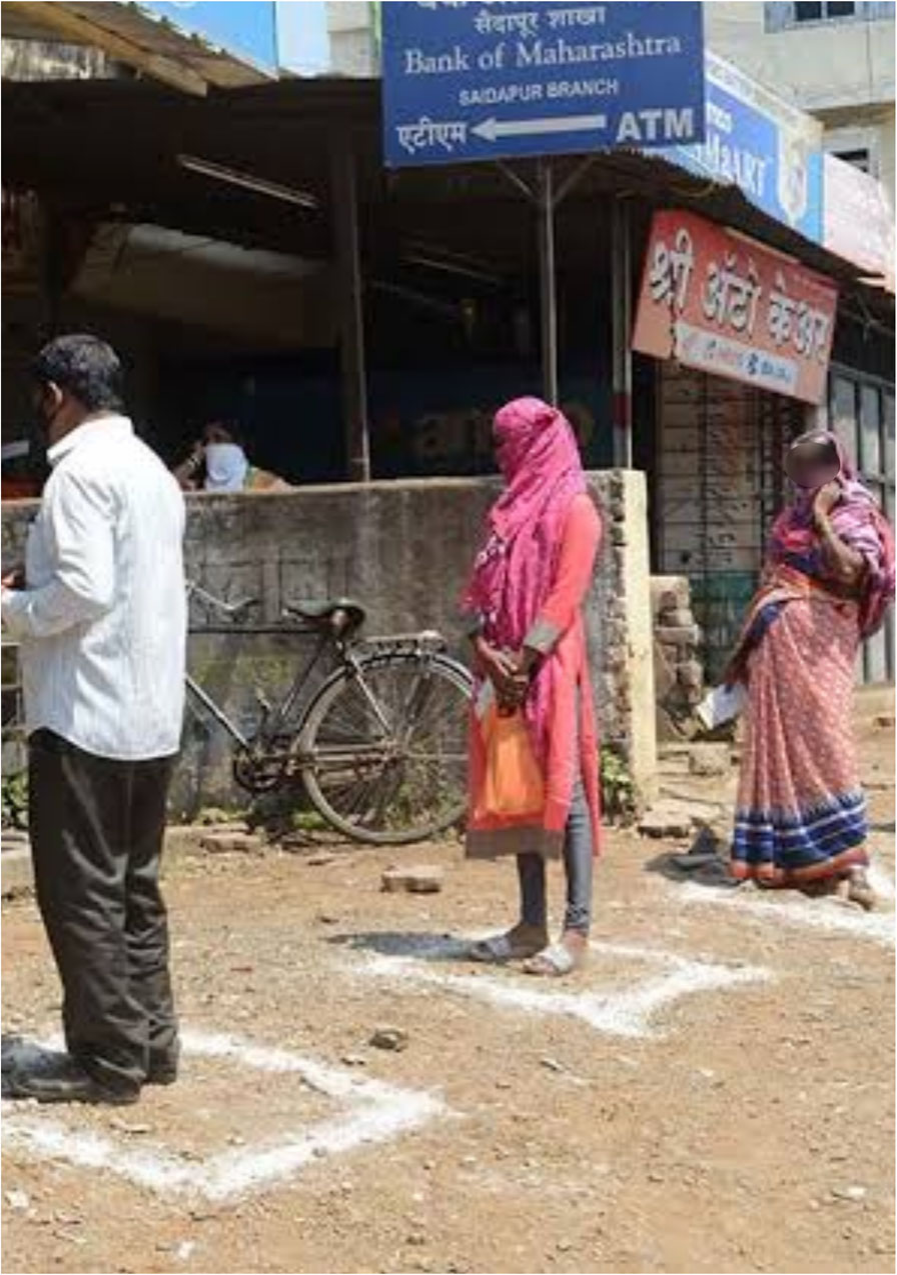
Photo taken by a male in Mumbai, India of people practicing social distancing measures in India

**Fig. 12:**
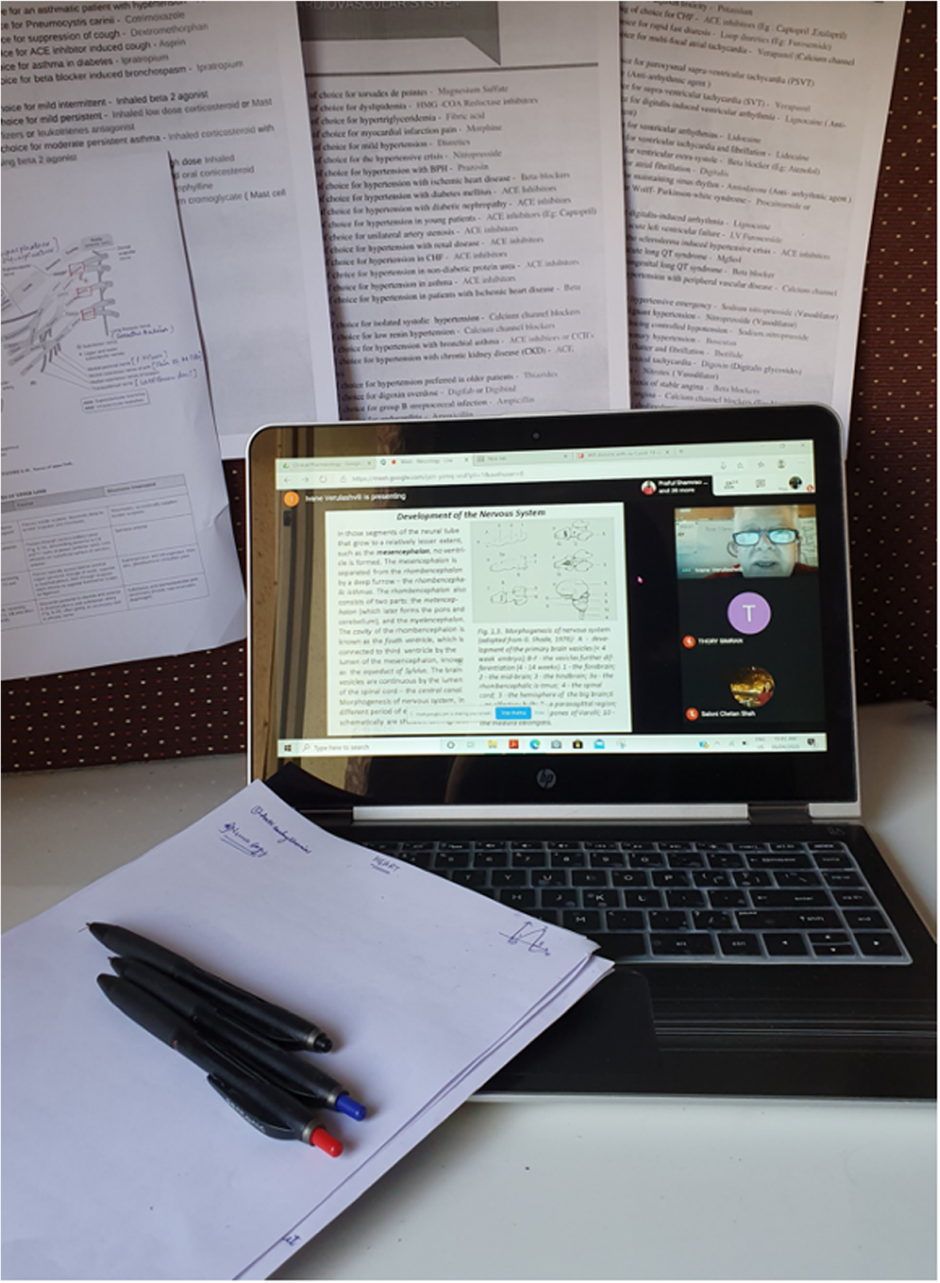
Photo taken by a female in Lahore, Pakistan of her virtual workspace for school

Analysis revealed four factors that transcended all levels of influence described by the participants. These factors were the new normal, future, time, and morbidity and mortality. A number of medical students found themselves adjusting to the new normal, which included changes in routines or schedules, changes from the old norm, or unusual situations that are now commonplace (Figs. 11 and 12).

“Grocery stores are now open, but we have to maintain social distancing.” (Insert figure 11 here, Photo taken by a male in Mumbai, India of people practicing social distancing measures in India.)

“We used to go to college and study, but now our whole education has been limited to this device ‘laptop.’” (Insert Fig. 12 here, Photo taken by a female in Lahore, Pakistan of her virtual workspace for school.)

Many learners felt uncertain about the future.

*“The whole world changed in 2 months, we were waiting for Liverpool to win the premier league, everyone was sure of that, but look it's still suspended, and no one knows what will happen.” (Male – Cairo, Egypt)*

Life during COVID-19 also left learners with extra time on their hands.

*“I have a lot of extra time now that I don't have anywhere to be other than home. With this time, I have taken up new hobbies like baking as it distracts me from thinking too much about all that's going on in the world.” (Female – Dublin, Ireland)*

Some utilized this time to form new hobbies.

*“With this time, I have taken up new hobbies like baking as it distracts me from thinking too much about all that's going on in the world.” (Female – Dublin, Ireland)*

**Fig. 13:**
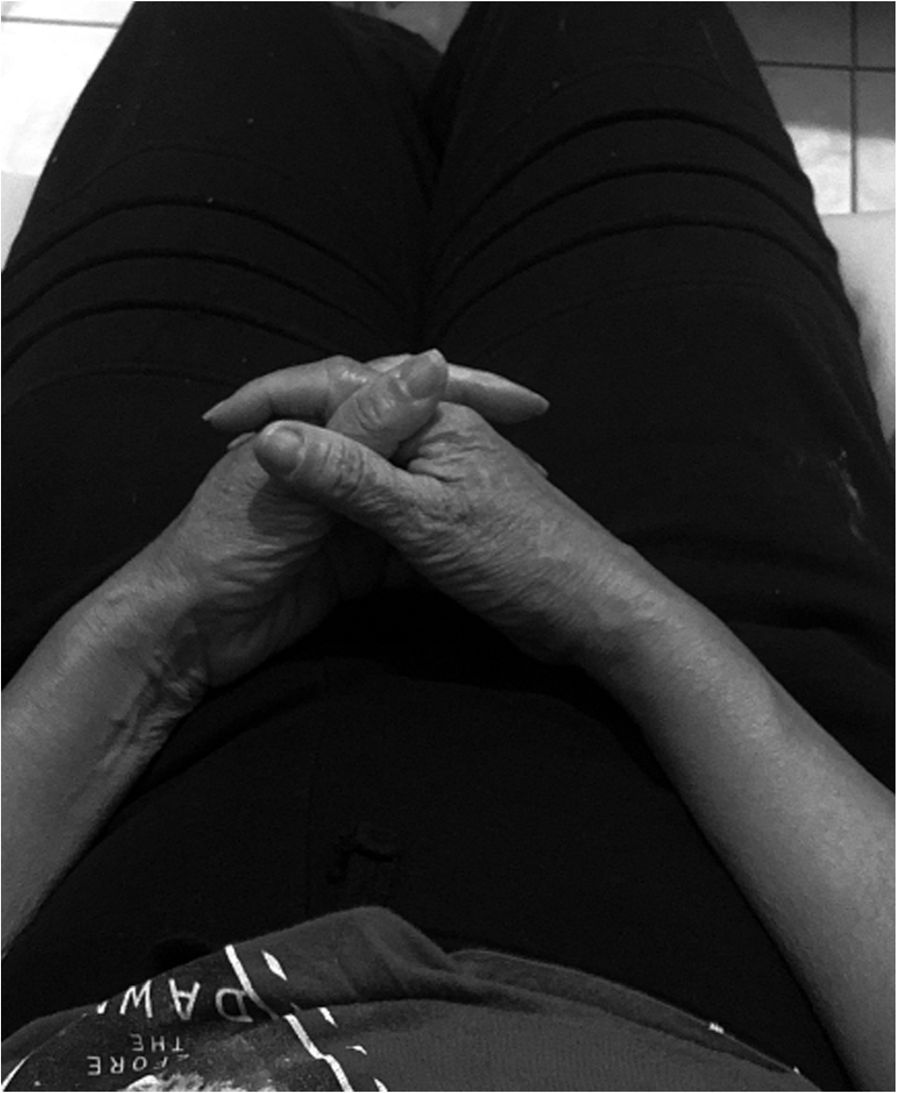
Photo taken by a female in Mexico City, Mexico of her mother’s hands

Participants noted that they were seeing or hearing about death and illness in the healthcare setting, but they were also seeing it in their personal lives and in their communities. One participant shared a family photo and explained that safety restrictions made it hard to process loss (Fig. 13).

*“These are my moms’ hands while she was informed that her big brother had died a couple of hours ago because of COVID-19. Because of the health situation, she wasn’t able to see his body or to even have a moment of grief in the funeral. This picture for me represents the dark side of this pandemic, represents the fact that death can occur to everyone in any context.” (Insert Fig.* *13**here, Photo taken by a female in Mexico City, Mexico of her mother’s hands.)*

## Discussion

The COVID-19 pandemic is an ongoing global event with unprecedented impact on individuals and communities. As future physicians, medical students maintained a unique view of the world as the pandemic unfolded. This qualitative study utilized a modified photo-elicitation approach to capture the global impact of the COVID-19 pandemic on the lives of medical students and their communities around the world. The research team was comprised of students from across the globe in order to ensure truly equitable global health research. The photo-elicitation findings can be summarized using an explanatory model, which reviews the effect of COVID-19 at the individual, interpersonal, educational and societal levels. The overarching themes of the new normal, future, time, as well as morbidity and mortality were central components at each of these levels.

As individuals, medical students involved in the study faced emotional distress, fear, hopelessness, grief, and denial, leading to increased levels of stress and anxiety. These findings were consistent with research that found students to feel hopeless, exhausted, and emotionally unresponsive in quarantine [25]. Similar findings of increased stress were reported in university students during previous epidemics, such as the Severe Acute Respiratory Syndrome (SARS) outbreak in Hong Kong [26, 27]. Despite the emotional distress and mental health effects of the pandemic, students fought to stay optimistic and take advantage of their time. They developed adaptive coping mechanisms by engaging in physical activity and establishing routines in order to create a new normal. This appears to be a universal trend [28]. Students in Belgium and Indonesia were found to have developed both problem-focused (adapting to new learning approaches, seeking peer support) and emotional-focused (developing positive outlooks, seeking closer relationships with their community and religious organizations) strategies similar to the students involved in this study [26, 28]. Previous studies have emphasized the importance of these actions for alleviating both acute and long-term stress [29, 30]. The importance of self-care and how students individually optimize their mental health is a critical lesson given recent reports of high physician burnout and suicide. The COVID-19 pandemic may provide students with the opportunity to discover how to deal with emotional distress and develop coping skills required to work in high stress environments. As one individual stated, “[when I was feeling overwhelmed] I decided to give myself a break and go to the stairs and breathe a little fresh air”. Recognizing one’s own emotional state and how to regulate it is essential for medical students as they learn to work in the high stress medical environment. Educators can help students by incorporating reflection in assignments and having open conversations about wellness [31].

The implementation of social distancing measures greatly impacted the interpersonal lives of our study participants. Many students were forced to move home with their parents where they may not have lived for many years. Other students remained with close friends but were separated from typical support networks. This study found that some participants appreciated the time they had with their family, while others struggled with isolation, separation, and stressors that the pandemic put upon their relationships. Concerns for the health and safety of participants’ families increased the feelings of isolation imposed by quarantine. Similar trends of increased anxiety and depression in students were reported during the SARS and Ebola epidemics [32, 33]. The use of telecommunication applications may be helpful in mitigating loneliness, maintaining interpersonal relationships, and comfort individuals concerned for their loved ones [34]. Institutions are recommended to make telecommunication apparatuses available for students and help students stay connected. For those geographical areas with barriers to telecommunication, mobilization of counselors and community health workers will be necessary to assist students in coping with distance measures.

Reflections from study participants revealed that the recurrent themes of isolation and emotional distress at the individual and interpersonal levels were somewhat abated by educational initiatives. Most classroom-based learning was moved online, which students were able to adjust to fairly well. Education served as one of the few constants during the pandemic and helped students stay connected as well as establish routines. Educators should be aware of the impact of structured lesson planning and adjust course work accordingly. Students may benefit from additional group-based assignments that utilize social networks oppose to individual work. These activities reduce feelings of isolation and help develop support structures for students struggling in quarantine [35]. Educators, however, should keep in mind that a shift towards online learning can create disparities in access. Several students experienced difficulties with obtaining reliable internet which created additional stress. This must be considered as we see a rise in online learning [13].

More advanced students were particularly concerned about lost or altered clinical experience and the impact of this on their futures. Many students were not permitted in hospital settings while those that were faced additional challenges. Studies have found that a decrease in patient-facing activities and separation from colleagues has caused a disruption in the interpersonal growth of physicians-in-training [36]. Socialization is key to the development of professional identity, defined as “the attitudes, values, knowledge, beliefs and skills shared with others within a professional group.” [36] Educators can combat this disruption by utilizing guided reflection exercises in which students are challenged to review their current roles, discuss strategies of wellbeing, and pursue ethical venues for volunteering [27, 31]. Additionally, implementation of case-review series or including students in telehealth medicine may help compensate for missed clinical time [14]. Faculty development is needed to help professors tailor curriculum to meet these changing needs [14].

The societal implications of the COVID-19 pandemic were a central topic of concern amongst many students. In this study sample, economic issues were repeatedly brought up as a major societal stressor. The current pandemic is unprecedented and the true ramifications on the economy are difficult to predict. The United Nations World Economic Situation and Prospects estimates a $8.5 trillion loss in output over the next 2 years [37]. During the SARS pandemic in 2003, models predicted that SARS would have catastrophic effects on the global economy [38]. However, it was found that the scale of the SARS impact on affected economies was far smaller than suggested by contemporary media reports and model estimates [38]. The ability to control and contain the SARS pandemic helped avoid more serious ramifications. Unfortunately, COVID-19 has not been contained in a similar manner and initial economic predictions may fall short of the prolonged impact of the pandemic. Students struggling with loans are particularly vulnerable at this time. Students also felt a strong desire to help their communities. Although their capacity as physicians may be limited, students can play a vital role in public health initiatives and educating their communities about safe practices [31].

This study was designed in part to create an opportunity for medical students around the globe to work together to explore the impact of COVID-19. The research team was composed of international medical students from 6 countries engaged in an equitable global health program. Although global health interest has increased over the past decade, opportunities for bi-directional learning are limited [39]. Students in low- and middle-income countries (LMICs) often struggle to find opportunities to engage in international collaborations. Research has shown that only 35% of authors on global health related studies were from LMICs [40].This study brought together medical students from six countries to work together on all aspects of project development. The primary investigators (R.F. and M.D.) conducted virtual workshops, led group discussions, and provided supplemental material to create an applied learning experience for the entire study team.

Our research should be interpreted in light of its limitations. This study was conducted between April and May of 2020 while the COVID-19 pandemic was still evolving rapidly. Findings and reflections may have changed over time as new information on the virus was learned. Additionally, while students from 13 countries were included in this study, they do not represent the ideals or behaviors of their respective countries as a whole. Trends were identified amongst students but cannot be generalized to their communities or to countries not included in this study. Finally, there are inherent limitations to self-reported data that stem from various social and psychological factors, including social desirability bias, which could distort photo-elicitation reflections in this study. Future studies examining long term sequela should be conducted to determine the impact of altered learning approaches on student development. Studies comparing new online modules are needed as education shifts towards more electronic based programs. Additionally, wellness programs should be evaluated in order to promote mental health amongst students.

## Conclusion

Among participating medical students the COVID-19 pandemic has impacted lives on multiple levels. As individuals, students felt emotional distress, but found resilience through problem-focused and emotional-focused coping mechanisms. Many students felt isolated as their interpersonal relationships were confined due to social distancing measures. These feelings could be combated with new educational initiatives focused on group collaboration and reflective assignments. Lastly, students had concerns with the economic ramifications of the virus and its impact on their future. This study brought together students from several different countries to engage in an applied learning program as a model for equitable global health research.

## Supplementary Information

**Additional file 1.**

## Data Availability

The datasets used and/or analyzed during the current study are available from the corresponding author upon reasonable request.

## Abbreviations

COVID-19: SARS-CoV-2
PHI: Public health interventions
MPH: Master’s in public health
GEMx: Global educational exchange in medicine and the health professions
ECFMG: Educational commission for foreign medical graduates
REDCap: Research electronic data capture
SARS: Severe acute respiratory syndrome
LMICs: Low- and middle-income countries
