# Supplementary material for: Life during the pandemic: an international photo-elicitation study with medical students
Source: BMC Med Educ. 2021 Apr 27;21:244. doi: 10.1186/s12909-021-02684-x (PMC8078097; doi:10.1186/s12909-021-02684-x)
Supplement: Supplementary file 1 — Additional file 1. [file 12909_2021_2684_MOESM1_ESM.docx]

**Title**

Life During the Pandemic: An International Photo-Elicitation Study with Medical Students

Dworkin, M.^1-2^, Akintayo, T.^3^, Calem, D.^4^, Doran, C.^2^, Guth, A.^2^, Kamami, E.M.^5^, Kar, J.^6^, La Rosa, J.^2^, Lui, J. C.^7^, Peji, N.^8^, Frasso, R.^2^

1. Warren Alpert Medical School, Brown University, Providence, RI, United States
2. College of Population Health, Thomas Jefferson University, Philadelphia, PA, United States
3. Obafemi Awolowo University, Ife, Nigeria
4. Sidney Kimmel Medical College, Thomas Jefferson University, Philadelphia, PA, United States
5. Drexel University, Philadelphia, PA, United States
6. New Vision University School of Medicine, Tbilisi, Georgia
7. Royal College of Surgeons, Dublin, Ireland
8. Universidad Nacional Autónoma de México, Facultad de Medicina, Mexico

Corresponding author - Rosemary Frasso

Survey

1. How old are you?
2. What gender do you identify with?

- Female
- Male
- Other

1. What university are you affiliated with?
2. What city is your university located in?
3. What is the name of the degree you are currently pursuing?
4. How many years is your degree program?
5. What year of study are you currently in?
6. Please help us understand your current living situation. Where are you currently located?
7. Did your university ask you to relocate due to COVID-19?

- Yes
- No

1. Did you relocate because of COVID-19?

- Yes
- No

1. Who do you currently live with?

- Alone
- A partner
- Family
- Friends
- Other

1. How many hours per week did you have classes prior to the COVID-19 pandemic?
2. Are students at your university still participating in classes?

- Yes-in person
- Yes- online or virtually
- No
- Other

1. Were you participating in clinical rotations prior to the pandemic?

- Yes
- No

1. Are you participating in clinical rotations during the pandemic?

- Yes
- No

1. Has your academic timeline been changed? For example, has there been a change in your graduation dates or examination times? If yes, please describe.

- Yes
- No

1. Please provide 3-4 photos taken between May 2^nd^ and May15th to show daily life during the pandemic. Please write a short 1 paragraph reflection on the photograph including where you are and what you are doing at that time.
2. For each photo, please provide…

- Date photograph was taken
- General location photograph was taken
- Theme the photograph relates to
- Education
- Volunteering or community activism
- Daily life
- Mental Health
- Relationships
- Living Situation
- Other
- Describe and reflect on the photograph
- Share anything else you would like use to know
